# Supplementary material for: Wnt signaling enhances macrophage responses to IL-4 and promotes resolution of atherosclerosis
Source: eLife. 2021 Mar 15;10:e67932. doi: 10.7554/eLife.67932 (PMC7994001; doi:10.7554/eLife.67932)
Supplement: Supplementary file 1. [file elife-67932-supp1.docx]

qPCR primer sequences

| Target gene | Forward primer | Reverse primer |
| --- | --- | --- |
| *Axin2* | 5’-CAGCCCTTGTGGTTCAAGCT-3’ | 5’-GGTAGATTCCTGATGGCCGTAGT-3’ |
| *Socs1* | 5’-GACGCCTGCGGCTTCTATT-3’ | 5’-CAGCTCGAAAAGGCAGTCG-3’ |
| *Ccl17* | 5’-TACCATGAGGTCACTTCAGATGC-3’ | 5’-GCACTCTCGGCCTACATTGG-3’ |
| *Socs3* | 5’-TGAGCGTCAAGACCCAGTCG-3’ | 5’-CACAGTCGAAGCGGGGAACT-3’ |
| *Arg1* | 5’-CTCCAAGCCAAAGTCCTTAGAG-3’ | 5’-AGGAGCTGTCATTAGGGACATC-3’ |
| *Hprt* | 5’-GAGGAGTCCTGTTGATGTTGCCAG-3’ | 5’-GGCTGGCCTATAGGCTCATAGTGC-3’ |
| *Gapdh* | 5’-AGGTCGGTGTGAACGGATTTG-3’ | 5’-TGTAGACCATGTAGTTGAGGTCA-3’ |
| *Ptges* | 5’-GAAGAAGGCTTTTGCCAACCC-3’ | 5’-TCCACATCTGGGTCACTCCT-3’ |
| *Ptgs2* | 5’-AGCCAGGCAGCAAATCCTT-3’ | 5’-CAGTCCGGGTACAGTCACAC-3’ |
| *Hpgd* | 5’-TCATTAGCAGGGCTCATGCC-3’ | 5’-TGTGAATCCGATGATGCCGT-3’ |
| *Lef1* | 5’-TGTTTATCCCATCACGGGTGG-3' | 5’-CATGGAAGTGTCGCCTGACAG-3' |
| *Stat3* | 5’-CAATACCATTGACCTGCCGAT-3' | 5’-GAGCGACTCAAACTGCCCT-3' |
